# Adapted Smart-seq3xpress Facilitates Selective Microglial Transcriptomic Profiling From Frozen Brain Tissue

**DOI:** 10.1007/s10571-026-01743-5

**Published:** 2026-05-14

**Authors:** Dominika Dostalova, Pavel Abaffy, Eva Rohlova, Jan Kriska, Tomas Knotek, Jana Tureckova, Denisa Kirdajova, Miroslava Anderova, Lukas Valihrach

**Affiliations:** 1 Laboratory of Glial Biology and Omics Technologies, Institute of Biotechnology of the Czech Academy of Sciences, Vestec, Czech Republic; 2 Faculty of Science, Charles University, Prague, Czech Republic; 3 GeneCore Facility, Institute of Biotechnology of the Czech Academy of Sciences, Vestec, Czech Republic; 4 Department of Cellular Neurophysiology, Institute of Experimental Medicine of the Czech Academy of Sciences, Prague, Czech Republic; 5 Second Faculty of Medicine, Charles University, Prague, Czech Republic

**Keywords:** Enrichment, Frozen tissue, Microglia, PU.1, Single-nucleus RNA - sequencing, Stroke

## Abstract

Single-cell transcriptomics has revealed the central role of microglia in brain development, homeostasis, and disease, particularly in the context of neuroinflammation. While single-cell RNA-sequencing enables targeted microglial analysis from fresh tissue, studying these cells in cryopreserved or archival samples remains challenging due to the lack of a protocol for their specific enrichment and RNA-sequencing. In this study, we developed a method for profiling microglial nuclei from fresh-frozen tissue using Smart-seq3xpress. This approach relies on PU.1-based enrichment, made possible by a brief formaldehyde fixation step to preserve the antigen. To ensure compatibility with Smart-seq3xpress, we utilized a Thermolabile Proteinase K treatment to reverse cross-links, thereby maintaining high transcriptomic sensitivity. We benchmarked the method in a mouse model of ischemic stroke, evaluating both technical performance and its ability to capture biologically meaningful microglial states. Compared to standard single-nucleus protocols, our approach yielded higher gene and UMI counts and a greater proportion of coding reads. Transcriptomic profiles closely matched those from whole-cell RNA-sequencing, including the detection of activation markers and diverse microglial subpopulations. This approach enables high-resolution transcriptomic analysis of microglia from fresh-frozen or archival brain samples, overcoming major limitations of single-nucleus RNA sequencing. It broadens access to cellular insights from biobanked material, supporting both basic research and translational studies of neuroinflammatory disease.

**Graphical Abstract:**

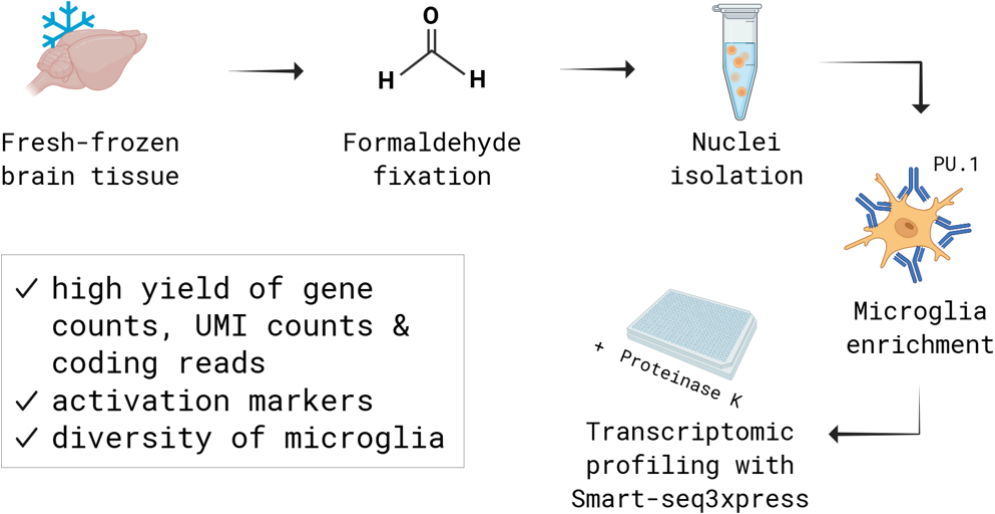

A modified Smart-seq3xpress protocol was developed for microglial profiling by combining formaldehyde fixation and PU.1-based nuclei enrichment from fresh-frozen brain tissue. The method yields high gene and UMI counts alongside strong coding coverage, enabling high-resolution characterization of diverse microglial activation states following stroke.

**Supplementary Information:**

The online version contains supplementary material available at 10.1007/s10571-026-01743-5.

## Introduction

Microglia, the resident immune cells of the central nervous system, play essential roles in brain development, homeostasis, and pathology. Single-cell (scRNA-seq) and single-nucleus RNA sequencing (snRNA-seq) have significantly advanced the capacity to study microglial heterogeneity and function (Hirbec et al. 2017). However, scRNA-seq requires fresh tissue, limiting its use for postmortem or biobanked human samples, which are predominantly available as frozen or fixed material.

snRNA-seq provides access to such preserved tissues but remains suboptimal for studying microglia. A common enrichment strategy in scRNA-seq relies on the surface marker CD11b, but this cannot be used for nuclei-based workflows, as nuclear membranes lack surface proteins. Without nuclear-specific markers, enrichment is not possible, resulting in data dominated by other cell types. As a result, snRNA-seq datasets often contain too few microglia for robust analysis, less than 10% (Zucha et al. 2024). This increases sequencing costs and reduces resolution, especially for rare or transient microglial states. Additionally, recent work has shown that snRNA-seq underrepresents many transcripts associated with microglial activation, including key immune and disease-related genes, calling into question its sensitivity for capturing cellular responses in pathology (Thrupp et al. 2020).

To overcome these limitations, we developed a protocol for profiling selectively enriched microglial nuclei using PU.1, a transcription factor localized to the nucleus and specific to the myeloid lineage (Smith et al. 2013). A brief formaldehyde fixation step stabilizes PU.1 detection (Nott et al. 2021), and crucially, the protocol is fully compatible with Smart-seq3xpress full-length RNA sequencing through the incorporation of Thermolabile Proteinase K (TLPK) for effective decrosslinking while preserving RNA integrity (Chung et al. 2022; Xu et al. 2023). Smart-seq3xpress combines high sensitivity with full-length transcript coverage, enabling isoform-level resolution and allele-specific analyses at single-cell depth (Hagemann-Jensen et al. 2022).

We benchmarked this protocol in a mouse model of ischemic stroke (Androvic et al. 2020), a condition characterized by pronounced microglial activation, and showed that our approach robustly captures both homeostatic and activated microglial states. By increasing microglial representation and preserving activation-related signatures, the protocol enhances both the technical performance and biological interpretability of snRNA-seq datasets. Its compatibility with archived tissue makes it broadly applicable to studies of neuroinflammation, neurodegeneration, and other brain disorders where microglia play a critical role.

## Methods

### Experimental Model-Middle Cerebral Artery Occlusion

Experiments were performed on 22 to 29-week-old C57Bl/6J male mice (Jackson Laboratory, Cat#000664). For each of the three isolation protocols, four mice were used: two subjected to permanent middle cerebral artery occlusion (MCAO) and two sham-operated controls. The mice were housed in an air-conditioned room (20–24 °C) under a 12-h light/dark cycle with ad libitum access to food and water, and in cages of 2–4 individuals. The method for inducing focal cerebral ischemia by permanent MCAO has been detailed previously (Androvic et al. 2020). Briefly, the left middle cerebral artery was permanently occluded at the distal segment using bipolar tweezers, and tissue samples were collected 7 days post-ischemia. Sham surgery was used as a control, and our previous results showed that sham surgery did not impact gene expression compared to naïve controls (Androvic et al. 2020).

### Isolation of Cortical Tissue From the Mouse Brain

Deeply anesthetized mice with a pentobarbital sodium solution (100 mg/kg, i.p.; Sigma-Aldrich, Cat#P3761; dissolved in 0.9% saline [Ardeapharma, registration number 76/224/95-C]) were perfused transcardially with cold (4–8 °C) isolation buffer (IB) composed of (in mM): NaCl 136.0 (Sigma-Aldrich, Cat#S9888), KCl 5.4 (Sigma-Aldrich, Cat#P5405), HEPES 10.0 (Sigma-Aldrich, Cat#H3375), glucose 5.5 (Sigma-Aldrich, Cat#G8270), with a pH of 7.4 and an osmolality of 290 ± 3 mOsmol/kg. Cortical tissue was isolated from the left hemisphere. The brain (+ 2 mm to −2 mm from bregma) was cut, and the sham or post‑ischemic parietal cortex was carefully dissected, avoiding the ventral white matter tracts. The experiments were not randomized or blinded. Tissue isolation was performed by all key personnel to ensure a consistent and accurate characterization of the ischemic lesion. To minimize bias, we followed standardized protocols and ensured that individuals from the same litter were distributed equally across all experimental groups.

### Dissociation of Brain Tissue Into

#### Single-Cell Suspension (LiveCells Protocol)

The freshly dissected cortex was placed in 1 mL of papain solution (20 U/mL, Worthington Biochemical Corp., Cat#LK003176; dissolved in sterile Earle’s balanced salt solution [EBSS, Worthington Biochemical Corp., Cat#LK003188]) containing 60 µL of Deoxyribonuclease I (240 U, Worthington Biochemical Corp., Cat#LK003170; dissolved in EBSS) and Actinomycin D (ActD; 25 µg/mL, Sigma-Aldrich, Cat#A1410-10MG; dissolved in dimethyl sulfoxide [Sigma-Aldrich, Cat#472301-100ML]). The tissue suspension was incubated at 37 °C for 30 min with shaking (ThermoMixer C, Eppendorf) at 1,000 rpm. During incubation, the tissue was triturated every 10 min by pipetting, first with a wide-bore P1000 tip (Axygen, Cat#TF­1005-WB-R-S), followed by a standard P1000 filter tip (Axygen, Cat#TF-1000-L-R-S). After incubation, the suspension was centrifuged (Centrifuge 5804R, Eppendorf) at 500 × g for 5 min at 2 °C, with the brake turned off and minimal acceleration.

The supernatant was carefully removed, and the pellet was resuspended in 1 mL of ice­cold resuspension buffer consisting of IB supplemented with an inhibitor solution (containing 1 mg/mL of ovomucoid protease inhibitor and 1 mg/mL of bovine serum albumin [BSA]; Worthington Biochemical Corp., Cat#LK003182; dissolved in EBSS), Deoxyribonuclease I (200 U), and ActD (2.5 µg/mL). The suspension was incubated for 5 min on ice. Subsequently, 1 mL of the resuspended cells was gently layered over 3 mL of an inhibitor solution. The samples were centrifuged at 200 × g for 7 min at 2 °C.

The resulting cell pellet was resuspended in 1 mL of preservation buffer (PB), consisting of 1% BSA (Gibco, Cat#15260037) in IB supplemented with ActD (2.5 µg/mL). The resuspended cells were then filtered through a 50 μm CellTrics filter (Sysmex, Cat#04­004­2327) and centrifuged at 300 × g for 5 min at 2 °C.

The supernatant was removed, leaving approximately 90 µL of the cell pellet in PB. Next, 10 µL of magnetically labelled anti-CD11b antibody (1:10 dilution, Miltenyi Biotec, Cat#130­093­634, RRID: AB_3718051) and 0.5 µL of Calcein Green (1:1000 dilution, Invitrogen, Cat#C34852) were added to the suspension, and the mixture was incubated at 4 °C for 15 min. After incubation, 1 mL of PB was added, and the sample was centrifuged at 300 × g for 5 min at 2 °C.

The supernatant was carefully removed, and the cell pellet was resuspended in 0.5 mL of cold PB before being transferred onto a 50 μm CellTrics filter placed on an MS magnetic column (Miltenyi Biotec, Cat#130-042-201) positioned on an OctoMACS Separator (Miltenyi Biotec, Cat#130-042-109). The column was washed four times with 500 µL of PB. After washing, the column was removed from the magnet, and cells were eluted using 2 × 1 mL of PB containing RNaseOUT Recombinant Ribonuclease Inhibitor (RNaseOUT; 0.2 U/µL; Invitrogen, Cat#10777019).

Shortly before sorting, 1 µL of Hoechst 33258 (Sigma-Aldrich, Cat#94403) was added to the suspension. Calcein Green^+^/Hoechst 33258^−^ cells were sorted into 384-well plates (Bio-Rad, Cat#HSP3801) containing lysis buffer (see Generation of Smart-seq3xpress libraries) (SFig. 1 A). All sorting was performed using a BD FACSMelody system (BD FACSChorus v1.3 software, BD FACS Aria Fusion, BD Biosciences) equipped with a 100-µm nozzle, with sample and plate cooling maintained at 4 °C and index sorting enabled. Immediately after sorting, the plates were promptly centrifuged and stored at − 80 °C.

#### Single-Nucleus Suspension (LiveNuclei Protocol)

The CD11b⁺ enriched single-cell population was centrifuged at 300 × g for 5 min at 2 °C and resuspended in 0.75 mL of lysis mix (10 mM NaCl, 3 mM MgCl₂ [Sigma-Aldrich, Cat#M1028], 0.10% [v/v] Nonidet P40 Substitute [Sigma-Aldrich, Cat#74385], 0.32 M sucrose [Sigma-Aldrich, Cat#84097], 1× cOmplete Mini protease inhibitor [Roche, Cat#11836170001], 10 mM Tris-HCl pH 7.4 [Sigma-Aldrich, Cat#T2194], 0.2 U/mL RNaseOUT). The suspension was transferred to a pre-chilled KIMBLE Dounce tissue grinder (Sigma-Aldrich, Cat#KT885300-0002), and nuclei were released using five strokes with Pestle B.

The suspension was filtered through a 30 μm CellTrics filter (Sysmex, Cat#04-004-2326) into a 2 mL DNA LoBind tube (Eppendorf, Cat#0030108078). The tissue grinder was rinsed with an additional 0.75 mL of lysis mix, and the wash was filtered into the same tube. The resulting suspension was carefully layered onto 0.5 mL of 1.2 M sucrose solution (containing 0.2 U/mL RNaseOUT; prepared in 10 mM NaCl, 3 mM MgCl₂, 10 mM Tris-HCl pH 7.4) and centrifuged at 5,000 × g for 20 min at 2 °C. The supernatant (both phases) was removed, and the pellet was resuspended in 1 mL nuclei wash buffer (Dulbecco’s Phosphate Buffered Saline [Sigma-Aldrich, Cat#D8537-500ML] supplemented with 0.5% BSA and 0.2 U/mL RNaseOUT). The suspension was filtered through a 30 μm CellTrics filter.

Before sorting, 1 µL of Hoechst 33258 was added to the suspension, and Hoechst 33258^+^ nuclei were sorted into 384-well plates containing lysis buffer (see Generation of Smart-seq3xpress libraries) using fluorescence-activated nuclei sorting (FANS) under the same parameters as described above (see Single-cell suspension [LiveCells protocol]) (SFig. 1B).

#### Fixed Single-Nucleus Suspension (FixedNuclei Protocol)

Freshly frozen brain cortex tissue was placed into 0.3 mL of chilled fixation solution (1% formaldehyde [Penta Chemicals Unlimited, Cat#14150 − 11000]; diluted in Dulbecco’s Phosphate Buffered Saline) in a 1.5 mL DNA LoBind tube (Eppendorf, Cat#0030108051) and disrupted using a pellet pestle (Fisher Scientific, Cat#12141364). The homogenized tissue was then transferred using a P1000 wide-bore tip into a 2 mL tube (Eppendorf, Cat#0030120094) containing 1.6 mL of fixation solution and shaken (Shaker SHO, Witeg) on ice at 300 rpm for 10 min.

Next, 0.1 mL of 2.5 M glycine (Sigma-Aldrich, Cat#50046) was added to the suspension, which was shaken again for an additional 5 min, followed by centrifugation at 1,100 × g for 5 min at 2 °C. The supernatant was carefully removed, and the tissue pellet was resuspended in 1 mL of ice-cold lysis solution (lysis mix without 1× cOmplete Mini protease inhibitor; see Single-nucleus suspension [LiveNuclei protocol]) using P1000 wide-bore pipette tips and centrifuged again at 1,100 × g for 5 min at 2 °C. This wash step was repeated to ensure thorough removal of residual fixation solution.

After the final centrifugation, the supernatant was removed, and the tissue pellet was resuspended in 1 mL of ice-cold lysis solution using P1000 wide-bore tips and transferred into a pre-chilled Dounce tissue grinder. Nuclei isolation was performed on ice with 5 strokes using Pestle A, followed by 10 strokes with Pestle B. The resulting suspension was filtered through a 30 μm CellTrics filter and incubated on ice for 30 min.

The entire suspension was carefully layered onto 0.5 mL of 1.2 M sucrose solution (see Single-nucleus suspension [LiveNuclei protocol]) and centrifuged at 5,000 × g for 20 min at 2 °C. After centrifugation, both phases were aspirated, and the pellet was resuspended in 1 mL of 1 M sucrose solution. The suspension was then layered onto 500 µL of fresh 1.2 M sucrose solution and centrifuged again at 5,000 × g for 20 min at 2 °C.

Both phases were carefully aspirated, and the nuclei pellet was resuspended in 1 mL of nuclei wash buffer. The sample was then centrifuged at 1,100 × g for 5 min at 2 °C. After discarding the supernatant, the pellet was resuspended in 500 µL of fresh nuclei wash buffer and incubated on ice for 30 min.

Fluorescently labelled antibodies were then added to the suspension—NeuN (1:500, Sigma-Aldrich, Cat#MAB377X, RRID: AB_2149209), PU.1 (1:500, Cell Signaling Technology, Cat#81886, RRID: AB_2799984), and ACSA-2 (1:200, Miltenyi Biotec, Cat#130­117­535, RRID: AB_2727978)—and incubated overnight on a rotating platform (MiniMixer™ 3D Nutating Shaker, Benchmark Scientific) at 4 °C. After incubation, the sample was centrifuged at 1,100 × g for 5 min at 2 °C. The supernatant was carefully removed, and the nuclei pellet was resuspended in 1 mL of nuclei wash buffer.

The suspension was filtered through a 30 μm CellTrics filter and prepared for FANS. Shortly before sorting, 1 µL of Hoechst 33258 was added to the suspension, and Hoechst 33258⁺/ACSA­2⁻/NeuN⁻/PU.1⁺ nuclei were sorted into of Smart-seq3xpress plates containing modified lysis buffer (see Generation of Smart-seq3xpress libraries) using FANS under the same parameters as described above (see Single-cell suspension [LiveCells protocol]) (SFig. 1 C). The full and comprehensive protocol has been deposited on protocols.io (Dostalova et al. 2025).

### Antibody Specificity

All antibodies were obtained from commercial sources, and verification of primary antibody specificity was based on the manufacturer’s technical specifications and established peer-reviewed literature. Specifically, the ACSA-2 antibody was validated for the astrocyte-specific antigen ATP1B2 via short hairpin RNA-mediated knockdown assays (Batiuk et al. 2017). For the NeuN antibody, the specificity was confirmed through genetic knockout of *Rbfox3* in mice (Wang et al. 2015). For CD11b MicroBeads and the PU.1 antibody, specificity was determined by the manufacturer’s internal validation protocols.

### Generation of Smart-seq3xpress Libraries

Libraries were prepared according to the published protocol (Hagemann-Jensen et al. 2022), following the specified parameters with minor modifications. Cells and nuclei isolated from the LiveCells, LiveNuclei, and FixedNuclei protocols were sorted into 384-well plates containing 0.3 µL of lysis buffer, dispensed into each well alongside 3 µL of silicone oil (100 cSt, Sigma-Aldrich, Cat#378364-250ML) using the MANTIS^®^ Liquid Dispenser (FORMULATRIX). The lysis buffer for LiveCells and LiveNuclei contained 0.5 mM dNTPs (Thermo Scientific, Cat#R0192), 0.125 µM oligo dTVN30 (IDT), 5% PEG8000 (Sigma-Aldrich, Cat#P1458-25ML), 0.0125 µL SIRV4 diluted 1:2000 (Lexogen, Cat#141.01), and 0.4 U RNaseOUT diluted in UltraPure DNase/RNase-Free Distilled Water (NFW, Invitrogen, Cat#10977035).

For the FixedNuclei protocol, the lysis buffer was adapted by incorporating 2.4 U/mL TLPK (New England Biolabs, Cat#P8111S) and diluting it in low-EDTA TE buffer (10 mM Tris, 0.1 mM EDTA; Invitrogen, Cat#12090-015) instead of NFW. Sorted cells and nuclei in the 384-well plates were promptly stored at − 80 °C until further processing.

After storage at − 80 °C, the plates were thawed and immediately incubated at 72 °C for 10 min, followed by reverse transcription and 15 PCR cycles for pre-amplification (C1000 Touch Thermal cycler, Bio-Rad), as outlined in the protocol. Post pre-amplification, the cDNA was diluted with 9 µL of NFW and stored at − 20 °C overnight.

The protocol for plates prepared from the FixedNuclei protocol was slightly modified. Initially, RNA-binding proteins were removed by TLPK during incubation at 37 °C for 30 min. The enzyme was subsequently deactivated by incubating the plates at 56 °C for 30 min, followed by RNA denaturation at 80 °C for 15 min. Before adding the reverse transcription mix, the plates were cooled to 4 °C, after which the standard protocol was resumed.

Subsequently, 1 µL of diluted cDNA from each well was transferred to prepared plates containing dried index primers (2.82 pmol, standard desalted oligo preps, IDT), followed by tagmentation using 0.002 µL of tagmentation DNA enzyme 1 per reaction (Illumina, Cat#15027865). Tagmentation was stopped by the addition of SDS (Invitrogen, Cat#15553027) to a final concentration of 0.2%.

Libraries were indexed by adding 5 µL of PCR mix containing Tween-20 (Bio-Rad, Cat#1662404) to a final concentration of 0.01% and amplified using 14 PCR cycles. The libraries were then pooled and cleaned using Sera-Mag Speed Beads (GE Healthcare, Cat#65152105050250) beads (0.7×) in 22% PEG8000, followed by a second clean-up using SPRIselect Beads (Beckman Coulter, Cat#B23318) at a 0.65× ratio.

The size of the prepared libraries was assessed using the Fragment Analyzer HS NGS Library kit (Agilent, Cat#DNF-474–0500), and concentrations were measured using Qubit. The sequencing was performed using 2 × 109 bp paired-end reads and 2 × 10 bp index reads (NovaSeq X Series 25B Reagent Kit [200 cycles]; Illumina, Cat#20125968). For the replicated experiment, a 2 × 159 bp sequencing run was performed using the NextSeq 1000/2000 P1 XLEAP-SBS Reagent Kit (300 cycles; Illumina, Cat#20100982) on a NextSeq 2000 System (Illumina).

### Data Analysis

#### Processing of Raw Data

FASTQ files, including index reads, were generated from cbcl-files using Illumina’s Bcl-convert (version 4.3.6) without index demultiplexing; all reads were exported as *Undetermined*. Low-quality bases were trimmed from Read 2 using TrimmomaticSE (version 0.39) (Bolger et al. 2014) with parameters “ILLUMINACLIP: SmartSeq3xpressAdapter.fa:2:30:10 LEADING:3 TRAILING:3 SLIDINGWINDOW:4:15 MINLEN:36”. Read identifiers from the filtered Read 2 dataset were extracted and used to retain only corresponding pairs from Read 1 and index reads (Index 1 and Index 2) using seqtk subseq (version 1.3-r106) (Li 2012). Filtered files were compressed with pigz (version 2.6) and re-evaluated with FastQC (version 0.11.9) (Andrews 2010).

The obtained files were processed using zUMIs according to the published protocol (Hagemann-Jensen et al. 2022), following their specified parameters. Briefly, using zUMIs (version 2.7.9e) (Parekh et al. 2018) using parameters specified in SFile 1 and conda environment included in zUMIs package with STAR (version 2.7.3a), mouse genome version GRCm38 and annotation gencode.vM8. Several downsampled matrices (1 × 10^4^, 2 × 10^4^, 5 × 10^4^, 1 × 10^5^ read pairs) were generated.

For downstream analysis, we selected a downsampling threshold of 20,000 reads per cell, as the differences between isolation protocols were notable at higher read counts. For the replicated experiment, we applied a downsampling threshold of 2,000 reads per cells for the lower sequencing depth.

#### Data Filtering

The data were further analyzed using the Seurat R package (version 5.0.1) (Hao et al. 2021). Initially, data from all samples underwent exclusion of genes associated ERCC RNA spike (ERCC-) and spatially inferred RNA velocity (SIRV). Individual isolation protocols were filtered according to percentage of spike (percent.spike), the number of detected genes (nFeature_RNA), the total RNA counts (nCount_RNA), the proportion of mitochondrial RNA (percent.mt), and the ratio of unique molecular identifier (UMI)-tagged reads to the total reads (UMIfraction). The cut-offs specific for each type of isolation protocol were the following: LiveCells—percent.spike < 80, nFeature_RNA > 1,000, nCount_RNA > 5,000, UMIfraction > 0.5; LiveNuclei—percent.spike < 75, nFeature_RNA > 600, nCount_RNA > 2, 000, UMIfraction > 0.5; FixedNuclei—percent.spike < 60, nFeature_RNA > 1000, nCount_RNA > 5,000, UMIfraction > 0.5. The cut-offs were determined based on the negative control, which did not contain any sorted cells or nuclei. For repeated experiment, the cut-offs specific for each type of isolation protocol were the following: LiveCells—percent.spike < 17, nFeature_RNA > 450, nCount_RNA > 700; FixedNuclei—percent.spike < 15, nFeature_RNA > 450, nCount_RNA > 500.

#### Normalization, Integration, and Clustering

For the initial analysis of all obtained cell populations, data were normalized using SCTransform and integrated with CCAIntegration. Visualization was performed using Uniform Manifold Approximation and Projection (UMAP) based on 50 principal components (PCs). Clustering was conducted using the FindNeighbors and FindClusters functions with a UMAP resolution set to 0.3. Cluster identification was carried out by evaluating the expression of well-established marker genes corresponding to expected cell populations.

To isolate a pure microglial population for downstream analysis, we performed post hoc transcriptional filtering using unsupervised UMAP clustering. Cluster was identified based on the enriched expression of canonical microglial markers (*Hexb*, *Cx3cr1*, *Siglech*, and *Trem2*), while clusters lacking these markers were excluded to ensure population specificity. The obtained dataset was normalized using SCTransform and integrated with CCAIntegration. UMAP visualization was generated using 50 PCs, followed by clustering with the FindNeighbors and FindClusters functions at a UMAP resolution of 0.85.

During this process, cluster 4 was excluded due to nonstandard cell characteristics (SFig. 2 A). In the LiveCells protocol, cells in this cluster appeared as doublets, suggesting potential cell aggregates. A scatter plot of forward scatter area versus forward scatter height was used to visualize and identify these doublets (SFig. 2B). In the LiveNuclei and FixedNuclei protocols, the excluded cluster exhibited abnormally high ribosomal content, which is atypical for nuclei preparations, suggesting cytoplasmic contamination or incomplete nuclear isolation (SFig. 2 C, D).

After excluding cluster 4, the microglia data were reprocessed using SCTransform and reintegrated with CCAIntegration. UMAP visualization was again generated using 50 PCs, followed by clustering with FindNeighbors and FindClusters at a UMAP resolution of 0.8. The refined microglia dataset was then used for all downstream analyses.

#### Identification of Cluster Markers

Normalized and scaled RNA assay data were used to identify marker genes in both the full dataset and the microglia subset using the FindAllMarkers function with the default Wilcoxon test. Significant markers were defined as those expressed in at least 80% of cells within a cluster (STab. 1, 2).

For all cell populations, subcluster identities were assigned by comparing the identified markers to known gene signatures from previously described cellular subtypes. Similarly, in the focused microglia analysis, subclusters were annotated using established microglial gene signatures from Sala Frigerio et al. (2019). Manual curation was applied in both cases to ensure accurate identification and characterization of the cellular subtypes.

The AddModuleScore function in Seurat was used to calculate module scores based on the top 10 marker genes for microglial subtypes from studies Sala Frigerio et al. (2019) and Garcia-Bonilla et al. (2024). The results were visualized using FeaturePlot to display the spatial distribution of subtype-specific gene expression across the UMAP embedding.

#### 3’ Bias Metrics

The BAM file with all samples was partitioned into three experimental conditions (LiveCells, LiveNuclei, and FixedNuclei) in a Linux environment (Ubuntu 22.04) by matching cellular barcodes performed from Seurat metadata using samtools (v1.16) and grep.

To differentiate between UMI-tagged and internal fragments, BAM files were filtered by their UB: Z tag status. Reads containing a valid DNA sequence (matching the UB: Z:[ACGT] pattern) were classified as UMI-tagged, while those with empty tags (UX: Z:\t) were categorized as internal. Spike-in sequences were independently extracted using header-matching for ERCC- and SIRV prefixes.

Gene body coverage was calculated using RSeQC (v5.0.3) (Wang et al. 2012) via the UseGalaxy.eu platform, utilizing the mm10_RefSeq.bed reference for genomic data and a custom-generated BED12 file (derived from the additional_sequence_annot.gtf) for spike-in sequences. A minimum mRNA length threshold of 100 bp was applied.

#### Distribution of cDNA Fragment Lengths

Insert size distributions were extracted from the template length field of mapped BAM files using samtools and awk. The absolute values of non-zero template lengths were visualized in R using density plots.

#### Differential Gene Expression Analysis

The differential gene expression analysis was performed to identify upregulated or downregulated genes in activated response microglia (ARM) compared to homeostatic microglia (HM; combination of homeostatic populations 1 and 2 – HM1 and HM2). Comparisons were conducted separately for each isolation protocol using Seurat’s FindMarkers function with the default Wilcoxon rank-sum test on normalized and scaled RNA assay data. Genes were considered differentially expressed if they were detected in at least 10% of cells in either group, with an absolute log2 fold change > 1 and an adjusted p-value < 0.05 to account for multiple testing (STab. 3).

#### Scatterplots

Mean normalized gene expression values were calculated for ARM cells across conditions. A scatter plot was generated to compare gene abundance between the LiveCells or LiveNuclei protocols (x-axis) and either the FixedNuclei isolation protocol (y-axis). Full results are provided (STab. 4, 5).

#### Gene Ontology Enrichment Analysis

Gene ontology (GO) enrichment analysis was conducted using the compareCluster function from the clusterProfiler R package (version 4.4.4) (Wu et al. 2021). The analysis was performed on genes with a log-normalized mean expression > 1 in both LiveCells and FixedNuclei datasets. The biological process ontology was selected for functional annotation. A significance threshold of adjusted p-value < 0.05 was applied to identify significantly enriched GO terms. This approach allowed for the comparison of functional pathways between the two conditions, highlighting key biological processes associated with each dataset (STab. 6).

#### Cell–Cell Communication Analysis

Cell–cell communication was analyzed using the CellChat R (v1.6.1) package on normalized and scaled RNA assay data (Jin et al. 2021). Preprocessed CellChat objects for LiveCells, LiveNuclei, and FixedNuclei were loaded and merged using mergeCellChat. Functional network similarities were computed with computeNetSimilarityPairwise and visualized using netEmbedding. Clustering was performed with netClustering to identify similar signaling patterns. The final merged object was saved for further analysis.

## Results

### PU.1-Based Enrichment Enhances snRNA-seq Profiling of Microglia in Frozen Brain Tissue

We developed a FixedNuclei protocol to profile selectively enriched microglial nuclei from fresh-frozen brain tissue using the transcription factor PU.1 as a nuclear marker. To preserve the PU.1 epitope for reliable detection, a brief formaldehyde fixation step was introduced, followed by nuclei isolation under mild conditions. The protocol was optimized for compatibility with full­length single-cell transcriptomic profiling using Smart-seq3xpress (Hagemann-Jensen et al. 2022), including an additional lysis step with TLPK (Chung et al. 2022) to reverse cross-linking while preserving RNA integrity (Methods).

**Fig. 1:**
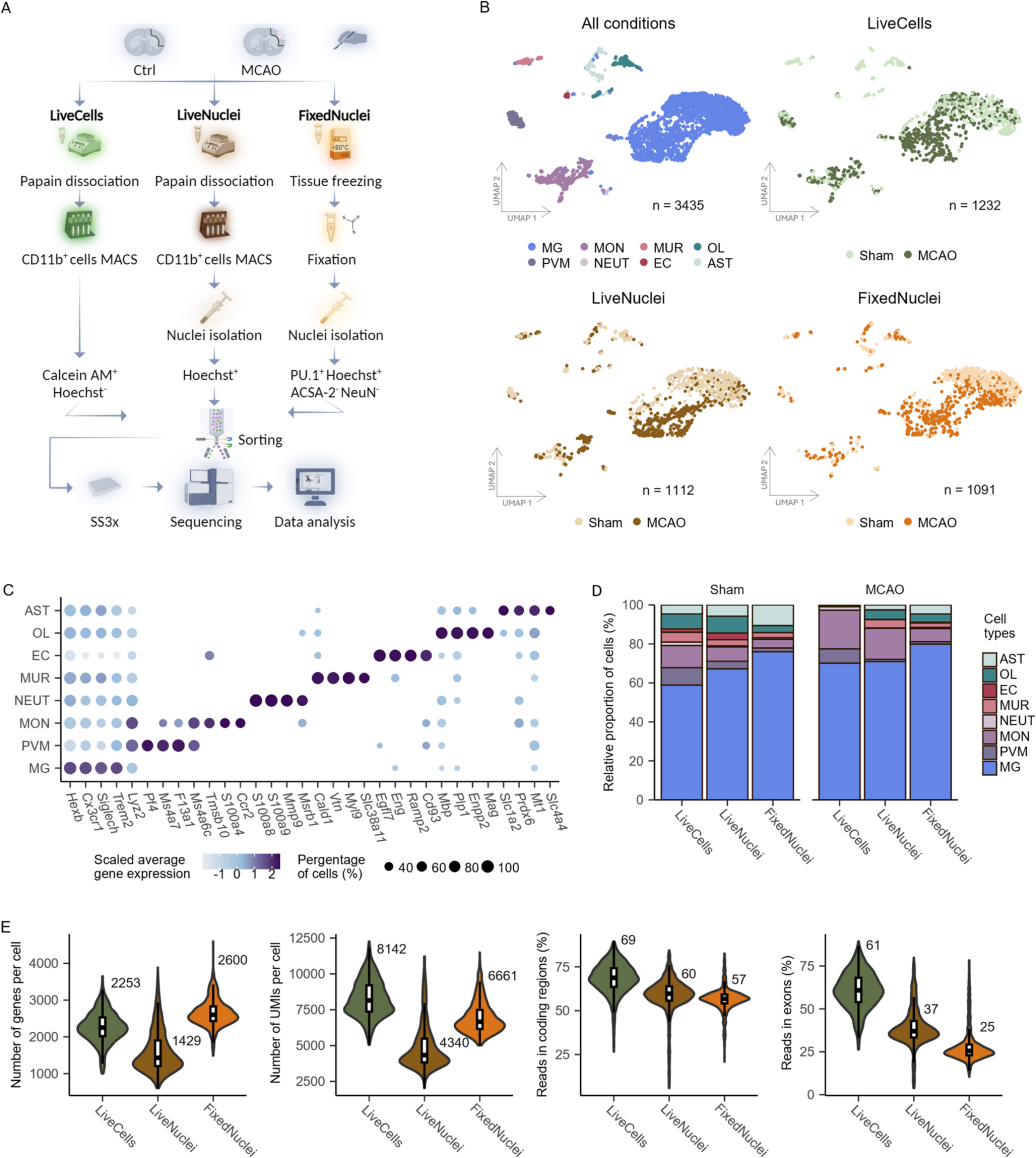
Comparison of three enrichment protocols for microglia populations. **A** An overview of the experimental pipeline: Tissue was processed using three protocols (LiveCells, LiveNuclei, FixedNuclei) for the enrichment of microglia populations. Isolated cells were sorted into 384-well plates for RNA-seq library preparation using the Smart-seq3xpress approach, followed by downstream analysis (Created with BioRender.com). **B** UMAP visualization showing identified clusters across all conditions: microglia (MG, *n* = 2 420), monocytes (MON, *n* = 387), perivascular macrophages (PVM, *n* = 144), oligodendrocytes (OL, *n* = 162), astrocytes (AST, *n* = 156), mural cells (MUR, *n* = 97), endothelial cells (EC, *n* = 36), and neutrophils (NEUT, *n* = 33). Cluster representation across isolation protocols (LiveCells, LiveNuclei, and FixedNuclei) and conditions (Sham vs. middle cerebral artery occlusion; MCAO) is included. **C** Dot plot showing canonical marker genes used for cell cluster identification. **D** Stacked bar plot representing the relative proportions of cells across isolation protocols (LiveCells, LiveNuclei, and FixedNuclei) divided into MCAO and Sham groups. **E** Violin plots with overlaid box plots summarizing technical parameters for the whole fraction of enriched cells: number of genes per cell, number of unique molecular identifiers (UMIs) per cell, reads in coding regions, and reads in exons for LiveCells, LiveNuclei, and FixedNuclei. Median values are displayed in the top-right corner of each plot. Box plots show medians (black line), first and third quartiles (white boxes), and whiskers extending to 1.5× the interquartile range

To evaluate this approach, we applied it to cortical tissue from a mouse model of MCAO, collected 7 days post-stroke, along with sham-operated controls. We compared the transcriptomic profiles generated using the FixedNuclei protocol to two established methods: (1) LiveCells – CD11b^+^ enriched cells by magnetic activated cell sorting from fresh-frozen tissue; and (2) LiveNuclei – nuclei isolated from the CD11b⁺-enriched cell suspension. While CD11b is widely used for isolating myeloid cells in live-cell workflows, it cannot be used for direct nuclear enrichment. In contrast, the FixedNuclei protocol enables targeted sorting of PU.1⁺ nuclei from lightly fixed, frozen tissue, allowing for selective microglial enrichment in a nuclei-compatible format (Fig. 1A).

Across all protocols, we identified eight major brain cell types: microglia, monocytes, perivascular macrophages, oligodendrocytes, astrocytes, mural cells, neutrophils, and endothelial cells (Fig. 1B). Microglia were characterized by high expression of canonical markers including *Hexb*, *Cx3cr1*, *Siglech*, and *Trem2* (Fig. 1C), and were the dominant population, comprising over 60% of the dataset (2,420 cells/nuclei) (Fig. 1D). We also note occasional detection of microglial marker genes in non-microglial clusters (Fig. 1C), which is likely a result of index hopping—a known technical artifact in sequencing that can lead to barcode misassignment, particularly when one cell type dominates the dataset (Costello et al. 2018).

To enrich microglia, we employed magnetic sorting based solely on CD11b expression, a commonly used myeloid marker. Unlike the CD11b⁺/CD45^low^ gating strategy often applied in FACS, our simplified approach reduces cell stress and enhances viability, helping preserve microglial transcriptomic integrity (Milich et al. 2021; Todd et al. 2021). However, CD11b is also expressed by other myeloid cells, which likely contributed to the presence of monocytes and perivascular macrophages in the LiveCells and LiveNuclei protocols. In contrast, the FixedNuclei protocol used a more selective nuclear marker-based strategy for sorting, resulting in higher microglial purity and reduced contamination from other lineages (Fig. 1D).

We next evaluated protocol performance based on key technical parameters. Surprisingly, the FixedNuclei protocol detected the highest number of genes per cell (2,600), surpassing both LiveCells (2,253) and LiveNuclei (1,429). Similarly, it yielded more UMIs per cell (6,661) than LiveNuclei (4,340), though not as many as LiveCells (8,142) (Fig. 1E). These trends were consistent across both MCAO and Sham conditions (SFig. 3A) and were largely reproduced in an independent experiment, except for slightly lower gene counts per cell in the FixedNuclei protocol (SFig. 3B). All comparisons were performed at a standardized sequencing depth of 20,000 reads per cell to maintain analytical consistency. At higher read depths (5 × 10⁴ and 1 × 10⁵), performance gaps became more pronounced, with no plateau observed (SFig. 3C), indicating true differences in RNA capture efficiency rather than coverage bias.

Read mapping statistics further reflected the expected differences between nuclear and whole-cell preparations. For coding regions, FixedNuclei achieved 57% of reads mapped—slightly lower than LiveNuclei (60%) and below LiveCells (69%). Reads mapping to exonic regions followed a similar trend: 25% for FixedNuclei, 37% for LiveNuclei, and 61% for LiveCells (Fig. 1E). These differences are expected, as nuclei contain higher levels of pre-mRNA and retained introns, whereas cytoplasmic RNA is enriched for fully spliced, exon-dense transcripts (Zheng et al. 2020).

Additionally, we evaluated the impact of fixation and TLPK on RNA quality. Analysis confirmed consistent RNA integrity across protocol conditions (SFig. 4A, B). The observed shifts in 3’ bias reflect the differences in the input material—whole cells and nuclei (SFig. 4A).

Together, these results demonstrate that our PU.1-based enrichment strategy preserves RNA integrity and improves the quality of nuclei-based transcriptomic data compared to standard snRNA-seq. While whole-cell protocols still offer the richest transcriptomic coverage, the FixedNuclei protocol enables robust profiling of microglial populations from frozen tissue—addressing the key limitations of marker availability, data sparsity, and RNA degradation in archival samples.

### PU.1-Based Approach Preserves Microglial Heterogeneity in Frozen Tissue

To assess how well microglial diversity is preserved across protocols, we performed unsupervised clustering of all microglia and analyzed the resulting transcriptional states. This allowed us to determine whether our PU.1-based FixedNuclei protocol captures biologically meaningful microglial heterogeneity comparable to established methods.

**Fig. 2:**
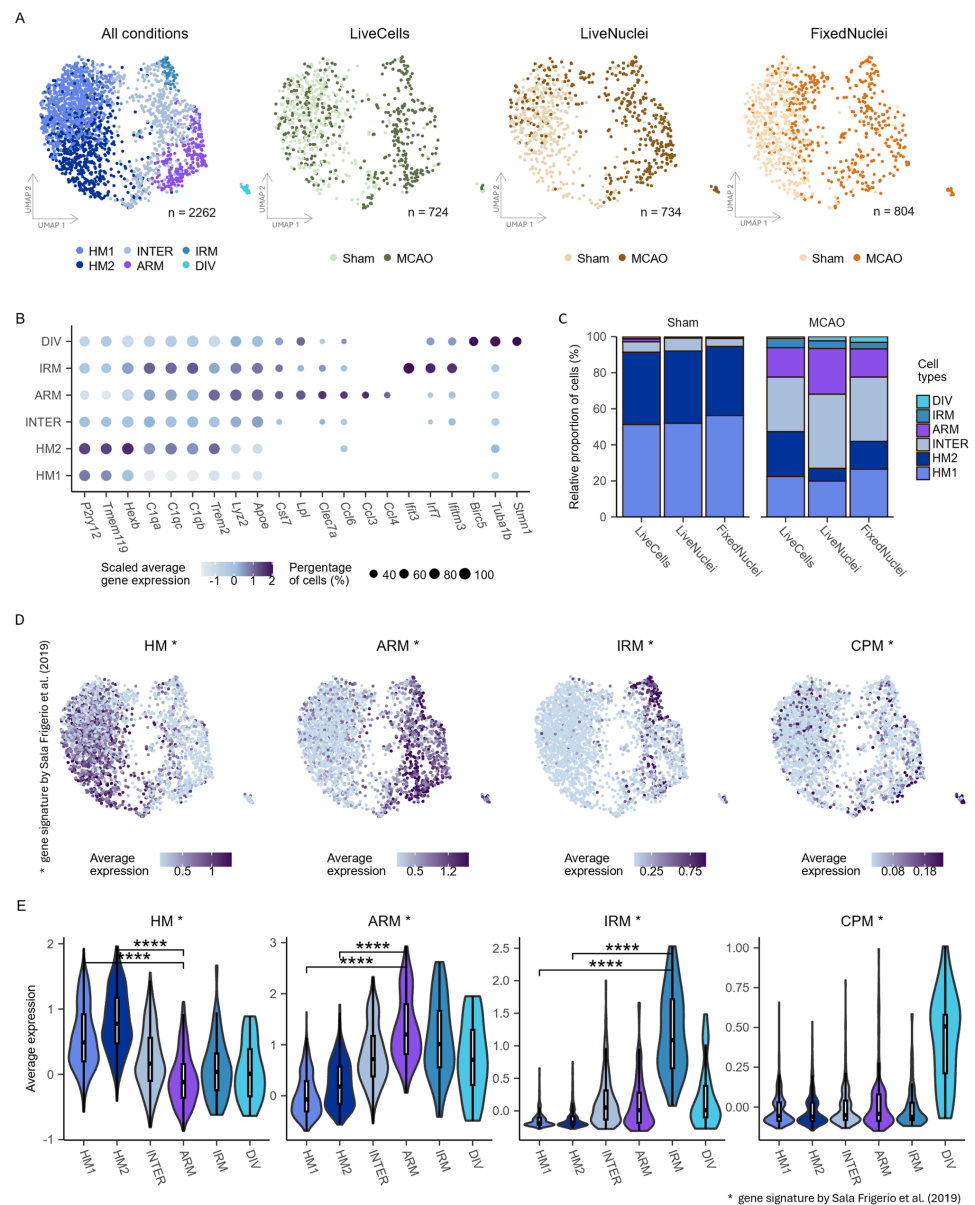
Characterization of microglia subpopulations. **A** UMAP visualization showing identified microglia clusters across all conditions, including homeostatic 1 (HM1, *n* = 841), homeostatic 2 (HM2, *n* = 609), intermediate (INTER, *n* = 489), activated response (ARM, *n* = 238), interferon response (IRM, *n* = 56), and dividing (DIV, *n* = 29) microglia. Additionally, the cluster representation is displayed for each isolation protocol (LiveCells, LiveNuclei, and FixedNuclei) in both Sham and middle cerebral artery occlusion (MCAO) conditions. **B** Dot plot showing canonical marker genes used for the identification of microglia subclusters. **C** Stacked bar plot representing the relative proportions of cells across isolation protocols (LiveCells, LiveNuclei, and FixedNuclei) divided into MCAO and Sham groups. **D**, **E** Evaluation of microglial subclusters using external signatures. **D** Feature plot visualizing the average expression of microglia gene signature as defined by Sala Frigerio et al. (2019) - homeostatic (HM), ARM, IRM, and cycling/proliferating (CPM) microglia. **E** Violin plots with overlaid box plots summarizing the average expression of microglia gene signature as defined by Sala Frigerio et al. (2019) across our microglia subpopulations. Box plots show medians (black line), first and third quartiles (white boxes), and whiskers extending to 1.5× the interquartile range. Statistical significance of differences in average gene expression between HM1, HM2 versus targeted populations was evaluated using an unpaired Wilcoxon rank-sum test

We identified six microglial subpopulations across all datasets: HM1, HM2, ARM, interferon response microglia (IRM), an intermediate state (INTER), and a small population of dividing microglia (Fig. 2A, SFig. 5A). HM1 and HM2 expressed canonical homeostatic genes such as *P2ry12* and *Tmem119*, with HM2 also showing elevated levels of *Hexb*, *C1qa*, *C1qb*, and *Trem2*. These populations were most abundant in sham samples but also remained detectable in post-stroke conditions. In contrast, ARM, IRM, and INTER subtypes were predominantly observed in MCAO samples. ARM cells expressed activation-associated genes including *Lyz2*, *Apoe*, *Cst7*, *Lpl*, *Clec7a*, *Ccl6*, *Ccl3*, and *Ccl4*. IRM cells were characterized by interferon response markers such as *Ifit3*, *Irf7*, and *Ifitm3*. The INTER population exhibited reduced homeostatic marker expression but lacked strong upregulation of activation markers (Fig. 2B, C).

To validate these classifications, we compared our cluster-specific gene signatures with those reported by Sala Frigerio et al. (2019) in a mouse model of Alzheimer’s disease. In their study, ARM and IRM states emerged gradually over several months in response to amyloid-β accumulation. In contrast, we observed similar microglial states within just seven days after stroke, highlighting the rapid response of microglia to acute injury. Marker genes from that study were highly enriched in our corresponding clusters, supporting the robustness of our classification (Fig. 2D, E). Furthermore, by assessing the transcriptional overlap with microglial clusters identified in a stroke model (Garcia-Bonilla et al. 2024), we observed that our ARM cluster primarily enriched for the Mg3, Mg4, and Mg7 signatures (SFig. 5B).

In summary, we identified six transcriptionally distinct microglial subpopulations across all protocols, capturing both homeostatic and activated states. All subtypes were consistently detected across protocols, demonstrating the preservation of microglial identity and transcriptional heterogeneity in our dataset. This highlights the robustness of our classification and confirms that the PU.1-based FixedNuclei method enables reliable profiling of microglia from frozen tissue.

### PU.1-Based Protocol Enables Detection of Inflammatory Microglial States

As key mediators of neuroinflammation, ARM are frequently studied in the context of neurological diseases. However, there is ongoing discussion regarding how isolation methods affect the detection of activation-related transcriptional changes (Thrupp et al. 2020). To address this, we compared the performance of our PU.1-based FixedNuclei protocol with the widely used LiveCells and LiveNuclei approaches.

**Fig. 3:**
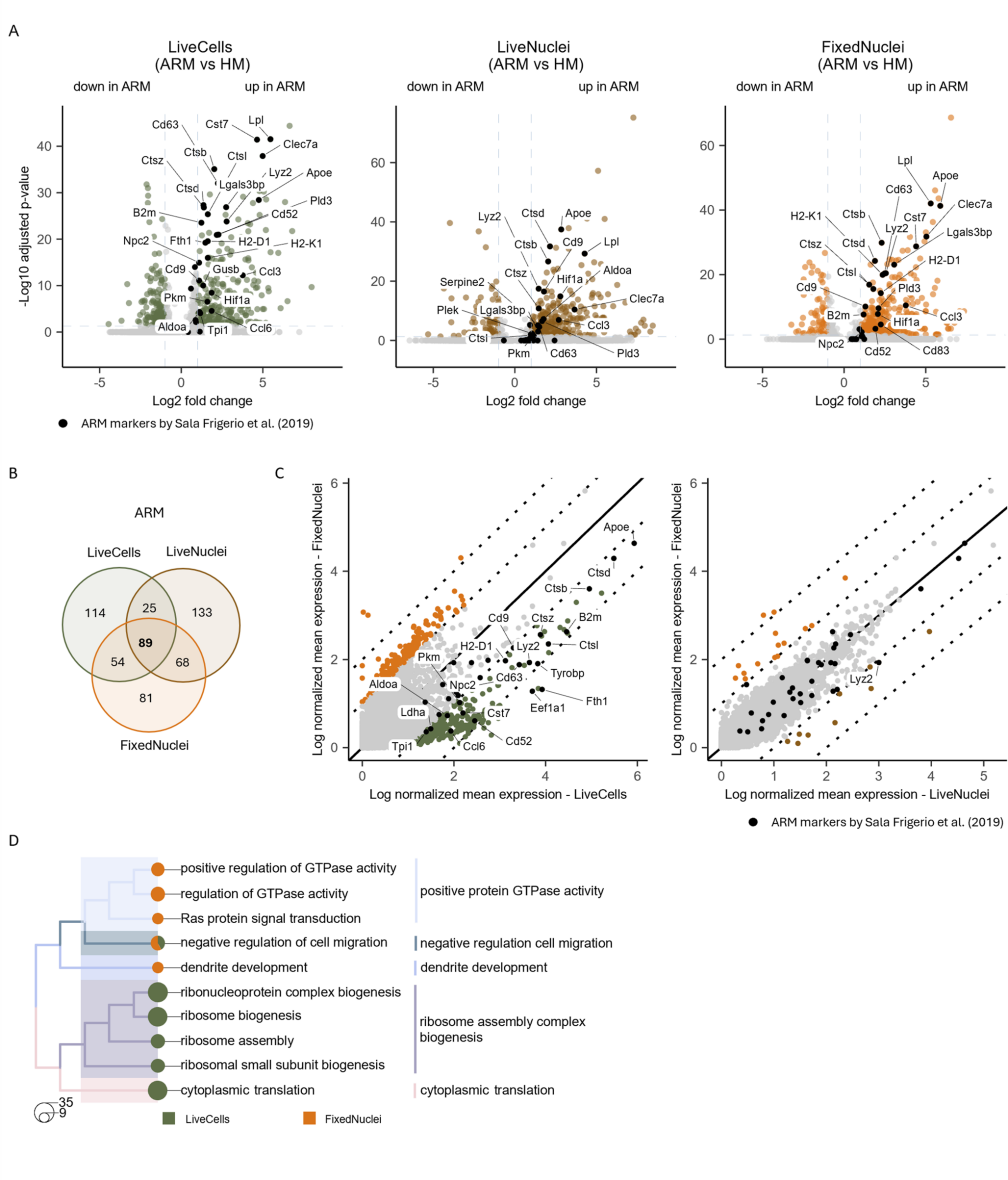
Comparison of microglia activation over isolation protocols. **A** Volcano plots illustrating differential gene expression of activated response microglia (ARM) compared to homeostatic microglia (combination of homeostatic 1 and 2; HM) across isolation protocols (LiveCells, LiveNuclei, and FixedNuclei). Each point represents the average value of a transcript. Color (green, brown, or orange) points indicate transcripts with an absolute log2 fold change > 1 and an adjusted p-value > 0.05 (− log10 scale). Highlighted dots (black) represent markers of the ARM population as characterized by Sala Frigerio et al. (2019), which were utilized here as a reference list for benchmarking our results, and significant genes are identified with gene names. **B** The Venn diagram illustrates the overlap of differentially expressed genes in the ARM population across the different isolation protocols. Differentially expressed genes were selected based on a log2 fold change > 1 and an adjusted p-value < 0.05. **C** Scatter plots showing the mean normalized gene abundance in the ARM population, comparing isolation protocols: LiveCells vs. FixedNuclei and LiveNuclei vs. FixedNuclei. The solid black line represents no fold change, while the dotted black lines denote 2- and 4-fold differences between isolation protocols. Genes described by Sala Frigerio et al. (2019) as activation markers are highlighted with black color to facilitate benchmarking, and genes with log-normalized mean expression > 1 are identified with gene names. Full results are provided (STab. 4, 5). **D** Tree plot of the top 10 significant gene ontology biological process terms identified by overrepresentation analysis, grouped into five clusters. The analysis includes genes with log-normalized mean expression > 1 identified from the differential expression analysis LiveCells vs. FixedNuclei (Fig. 3C, left). Full results are provided (STab. 6)

We first identified differentially expressed genes in ARM compared to HM population across all conditions (Fig. 3A). To benchmark our findings, we utilized the canonical microglial activation signatures established by Sala Frigerio et al. (2019) as a reference list to identify and highlight these markers within our own experimental results. A shared set of 89 differentially expressed genes was found in the ARM population across all three protocols (Fig. 3B), indicating that a common activation signature is consistently captured, regardless of the isolation method. This gene set includes canonical activation markers such as *Apoe*, *Lyz2*, *Cd63*, and *Clec7a*, associated with phagocytosis and immune activation, as well as *Lpl*, *Ctsb*, *Hif1a*, and *Ccl3*, which reflect metabolic adaptation and inflammatory signaling in activated microglia.

To assess how activation gene expression levels varied across protocols, we examined log₂ fold changes in ARM and visualized mean gene expression using scatter plots (Fig. 3C). ARM-associated genes showed generally higher expression in LiveCells compared to FixedNuclei, particularly for inflammatory and phagocytic markers. This pattern aligns with findings by Thrupp et al. (2020), who reported reduced detection of activation genes and translational machinery in nuclei compared to whole cells. In contrast, differences between FixedNuclei and LiveNuclei were smaller, suggesting closer alignment between the nuclear protocols.

Due to this high transcriptional similarity and the resulting insufficient number of differentially abundant genes between the two nuclear methods, we focused our functional analysis on the contrast between LiveCells and FixedNuclei. To explore the functional relevance of these differences, we performed GO enrichment analysis on genes enriched in each protocol. LiveCells showed upregulation of ribosome-related pathways, while FixedNuclei was enriched for processes such as GTPase regulation and dendrite development (Fig. 3D).

In summary, our PU.1-based FixedNuclei protocol effectively detects activated microglial states in frozen brain tissue. Although expression levels differ from whole-cell data due to the absence of cytoplasmic RNA, activated microglial gene programs—including those relevant to inflammation and disease—can be reliably studied using this approach.

### PU.1-Based Profiling Captures Cell–Cell Interactions Among Microglia

While the transcriptional activation of microglia in brain pathologies is well-documented, analyzing intercellular communication provides additional insights into how cell populations interact and coordinate their responses during disease progression. In this study, we focused specifically on communication pathways within microglial populations using the CellChat tool, which relies on transcripts associated with intercellular communication. Since previous benchmarks have demonstrated that single-nuclei and single-cell datasets show high transcriptomic concordance, they provide a comparable level of reliability for inferring signaling potential (Bakken et al. 2018; Wu et al. 2019). In addition, the cluster of dividing microglia was excluded from the analysis because of the low number of cells captured in the LiveCells protocol.

**Fig. 4:**
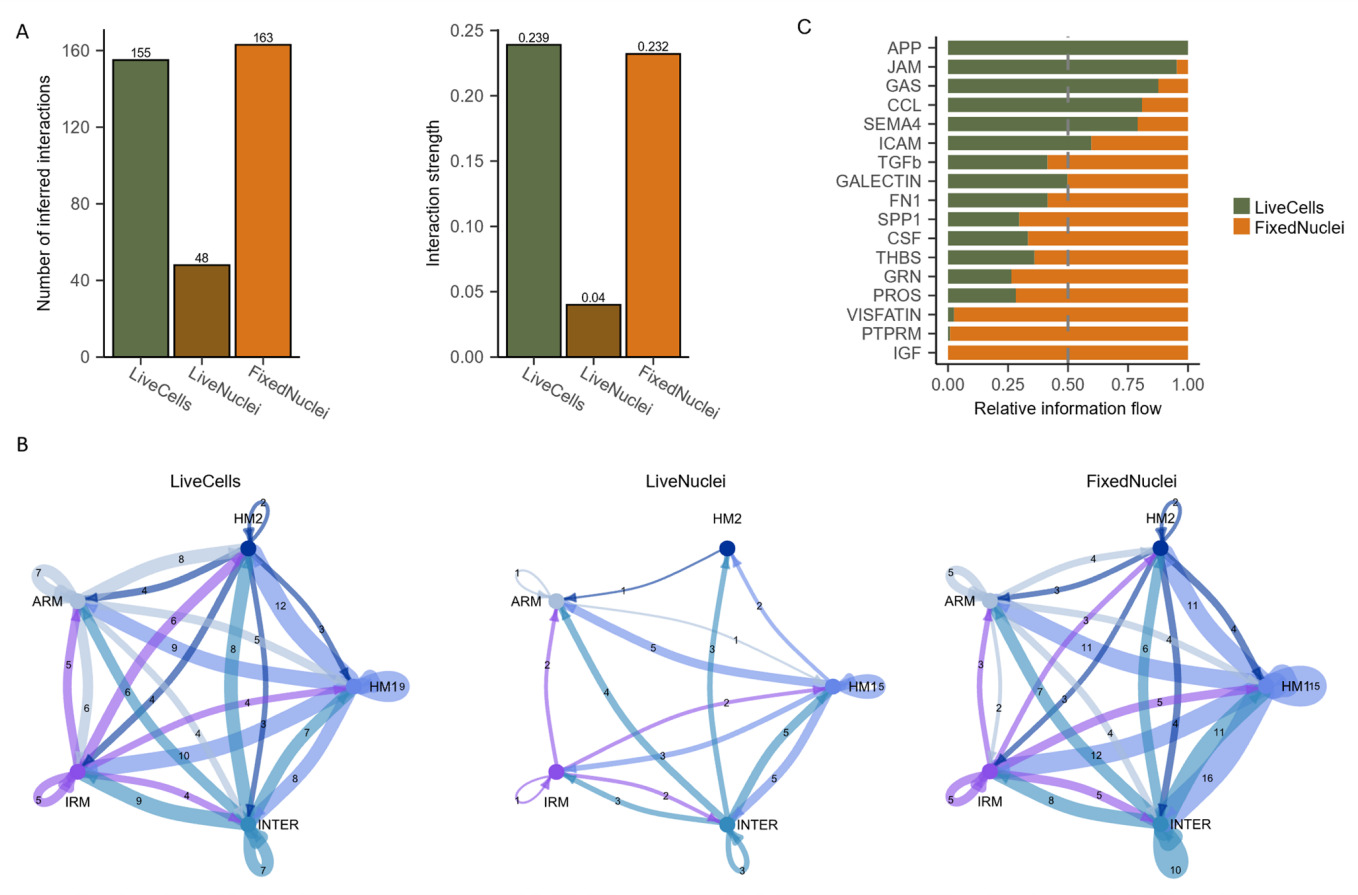
Cell–cell communication in microglial subclusters across isolation protocols. **A** (left) Number of predicted cell–cell interactions across different isolation protocols. **A** (right) Strength of predicted cell–cell interactions across different isolation protocols. **B** The number of significant ligand-receptor pairs between each pair of cell populations is represented, with edge width proportional to the total number of detected interactions. Thicker edges indicate a higher number of ligand-receptor pairs between the connected populations. **C** Significant signaling pathways were ranked based on differences in overall information flow, calculated by summing communication probabilities, to compare cell–cell communication across isolation protocols

We assessed communication using two quantitative metrics: the number of inferred ligand–receptor interactions and overall interaction strength. Both LiveCells and FixedNuclei protocols yielded a similar number of interactions (155 vs. 163), whereas LiveNuclei detected only 48 interactions (Fig. 4A, left). A comparable pattern was observed for interaction strength: LiveCells and FixedNuclei reached similar values (0.239 and 0.232), while LiveNuclei showed a fivefold lower value (0.04) (Fig. 4A, right). These trends were further illustrated in chord diagrams, which showed denser and more evenly distributed interactions in LiveCells and FixedNuclei compared to LiveNuclei (Fig. 4B). The reduced number of potential interactions in the LiveNuclei protocol (Fig. 4A, B) likely reflects the lower gene counts observed in this protocol (Fig. 1E), which may push specific signaling transcripts below the detection thresholds required by CellChat.

Given the poor performance of the LiveNuclei dataset, we excluded it from downstream pathway-level comparisons. Among the remaining protocols, we identified 17 signaling pathways, 15 of which were shared between LiveCells and FixedNuclei. Analysis across all identified pathways revealed expected components of microglial response to stroke, such as inflammatory pathways (e.g. CCL, ICAM, GAS) (Zhao et al. 2017; Müller 2019), clearance signals (e.g. SPP1, GRN) (Lui et al. 2016; De Schepper et al. 2023), and repair-associated signaling pathways that promote anti-inflammatory phenotypes (e.g., TGF-β, THBS) (Murphy-Ullrich and Suto 2018; Bedolla et al. 2024), tissue remodeling (e.g., FN1) (Bhattarai et al. 2024), and functional recovery (e.g. GRN, IGF) (Martens et al. 2012; Rusin et al. 2024). However, the relative information flow of these pathways varied between protocols (Fig. 4C), with several—including IGF, PTPRM, and VISFATIN—showing predominant detection in FixedNuclei, while pathways such as APP, JAM, and GAS were more prominent in LiveCells. This variation may reflect both technical biases and biological variability in the MCAO model.

In conclusion, the PU.1-based FixedNuclei protocol effectively captures microglial cell–cell communication networks and signaling dynamics, producing results that closely resemble those obtained from whole-cell protocols. Despite the inherent limitations of snRNA-seq—such as reduced detection of certain ligands or receptors—the PU.1-based approach enables meaningful inference of intercellular signaling and is well suited for studies using frozen or archived brain tissue.

## Discussion

Understanding microglial heterogeneity in pathological contexts requires not only sensitive transcriptomic techniques but also reliable enrichment strategies that preserve cellular diversity. Although CD11b is commonly used to enrich microglia from fresh tissue, its application to frozen samples has also been reported (Srinivasan et al. 2020). However, this approach often results in poor RNA quality, likely due to the challenges of processing fixed cell suspensions from thawed postmortem tissue. To address these limitations, we introduce a PU.1-based protocol, optimized for the isolation and transcriptomic profiling of microglia from fresh-frozen brain tissue, that is fully compatible with Smart-seq3xpress and provides a more reliable approach for studying these cells under disease conditions.

We selected PU.1 as the primary nuclear marker for microglial enrichment due to its stable expression across both homeostatic and reactive states. As a master regulator of microglial identity and function, PU.1 represents a more stable and reliable marker compared to alternatives like SALL1 and IRF8, whose expression fluctuates with activation status. Specifically, SALL1 is often downregulated in activated microglia (Buttgereit et al. 2016), whereas IRF8 is typically upregulated (Masuda et al. 2012).

Our approach is supported by previous findings from Alzheimer’s disease studies, where PU.1-based enrichment was successfully applied without fixation, allowing effective sorting and transcriptomic analysis (Mathys et al. 2019; Prater et al. 2023). The use of protease inhibitors during nuclei isolation in those protocols likely contributed to improved epitope preservation and antibody binding. Future comparisons between fixation and protease inhibition could help optimize epitope stability and overall protocol robustness.

Moreover, PU.1-based enrichment has also been applied to frozen brain tissue with mild fixation for microglial profiling, albeit using a different approach. In that study, SHARE-seq was implemented to simultaneously assess chromatin accessibility and RNA expression (Scholz et al. 2024). However, this method is less sensitive for transcriptome profiling compared to the Smart­seq3xpress technique used here, which achieves superior sensitivity thanks to its full-length transcript coverage.

Our protocol employed protein‑based enrichment by targeting the nuclear transcription factor PU.1; alternatively, microglial enrichment can be achieved using RNA‑based approaches. One such method is nuclampFISH, an amplified RNA FISH approach that enables cell sorting based on nuclear RNA expression and has been applied in downstream chromatin profiling (Liu et al. 2025). Another example is HCR-FlowFISH, which uses hybridization chain reaction amplification to detect specific transcripts and has been successfully applied to nuclei from frozen tissue for enriching rare cell populations in the context of PERFF-seq (Abay et al. 2025). RNA‑based enrichment is more labour-intensive and time‑consuming than our approach because it requires hybridization and signal‑amplification steps. However, it offers broader flexibility, as probe design can be tailored to selectively enrich the specific cell population of interest.

Moreover, the use of Smart-seq3xpress provided high-sensitivity and full-length transcript coverage, offering a significant advantage over 10x Genomics-based approaches. While we did not fully leverage the isoform-level resolution in this analysis—to ensure comparability with previous studies—Smart-seq3xpress remains a powerful platform for future investigations requiring greater transcriptomic depth, such as alternative splicing or low-abundance gene detection.

Despite these advances, our study has limitations. Biological variability introduced by the MCAO model may influence gene expression patterns, and the relatively low number of microglial cells and nuclei analyzed (2,262 total across three protocols) may limit the statistical power and depth of downstream analyses, increasing the potential for bias in interpretation. Furthermore, the limited number of biological replicates per condition restricted our ability to perform an analysis of inter-individual variability, such as through a pseudobulk approach.

In conclusion, our PU.1-based protocol offers a reliable method for enriching microglia from frozen brain samples, enabling high-quality transcriptomic profiling even in pathological conditions. This approach lays the groundwork for future studies aiming to resolve microglial diversity and function at single-cell resolution using full-length transcriptomic methods.

## Conclusion

In summary, we present a PU.1-based protocol for enriching microglia from fresh-frozen brain tissue, optimized for high-sensitivity transcriptomic analysis using Smart-seq3xpress. By targeting a nuclear marker with stable expression across microglial states, we achieved consistent enrichment suitable for profiling both homeostatic and activated populations. While our analysis was limited by biological variability inherent to the MCAO model and a modest number of captured cells, the results demonstrate the feasibility and reliability of this approach. This protocol provides a robust foundation for future studies of microglial heterogeneity in pathological conditions and offers a path toward leveraging full-length transcriptomic data to get deeper insights into microglial function and gene regulation.

## Supplementary Information

Below is the link to the electronic supplementary material.

Supplementary material 1 (ZIP 2245.5 kb)

Supplementary material 2 (ZIP 4520.9 kb)

## Data Availability

The data that support the findings of this study are publicly available on Zenodo (https://zenodo.org/records/16737457), and the code for analysis is available on GitHub (https://github.com/GliaOmicsLab/microglia-sc-snRNAseq). Data from the main experiment (all cells (https://scarfweb.nygen.io/eu-central-1/public/q2acwcw8) and the subset of microglia (https://scarfweb.nygen.io/eu-central-1/public/wxvqmskh)) are available for online analysis via the Nygen portal. A detailed version of the optimized protocol is available at Protocols.io (https://www.protocols.io/private/E618BD14711011F08A690A58A9FEAC02).

## Abbreviations

ARM: activated response microglia
BSA: bovine serum albumin
EBSS: Earle’s balanced salt solution
FixedNuclei: single-nucleus RNA sequencing from formaldehyde fixed tissue
GO: gene ontology
HM1: homeostatic microglia 1
HM2: homeostatic microglia 2
IB: isolation buffer
INTER: intermediate microglia
IRM: interferon response microglia
LiveCells: single-cell RNA sequencing from fresh tissue
LiveNuclei: single-nucleus RNA sequencing from cells obtained in LiveCells protocol
MCAO: middle cerebral artery occlusion
NFW: UltraPure DNase/RNase-Free Distilled Water
scRNA-seq: single-cell RNA sequencing
snRNA-seq: single-nucleus RNA sequencing
TLPK: Thermolabile Proteinase K
UMAP: uniform manifold approximation and projection
