# Supplementary figures and images for: Adapted Smart-seq3xpress Facilitates Selective Microglial Transcriptomic Profiling From Frozen Brain Tissue
Source: Cell Mol Neurobiol. 2026 May 14;46:113. doi: 10.1007/s10571-026-01743-5 (PMC13357468; doi:10.1007/s10571-026-01743-5)

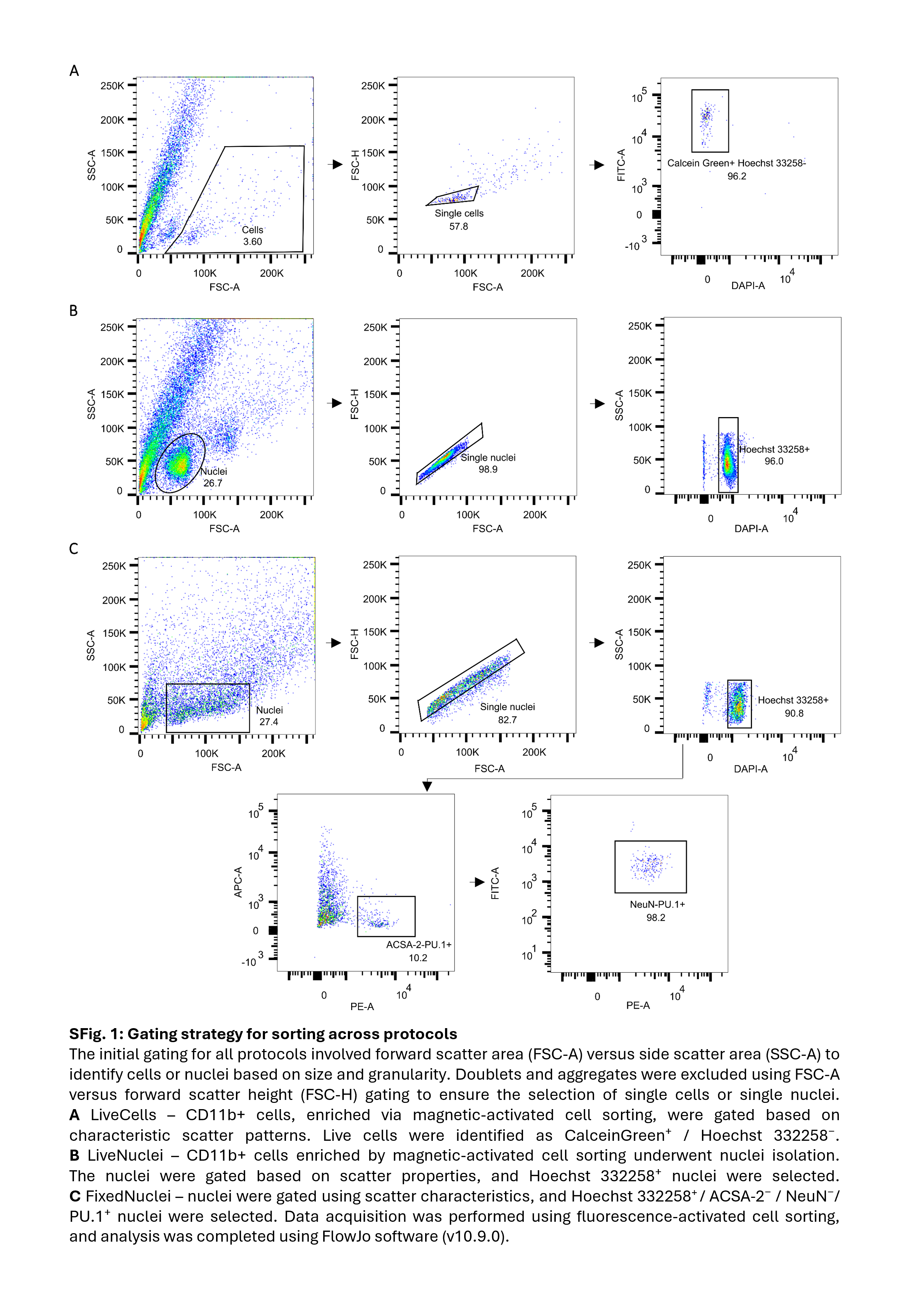

Supplement: Supplementary file 2 — Supplementary material 2 (ZIP 4520.9 kb) [file 10571_2026_1743_MOESM2_ESM.zip › SFig. 1_5/SFig. 1.tif]

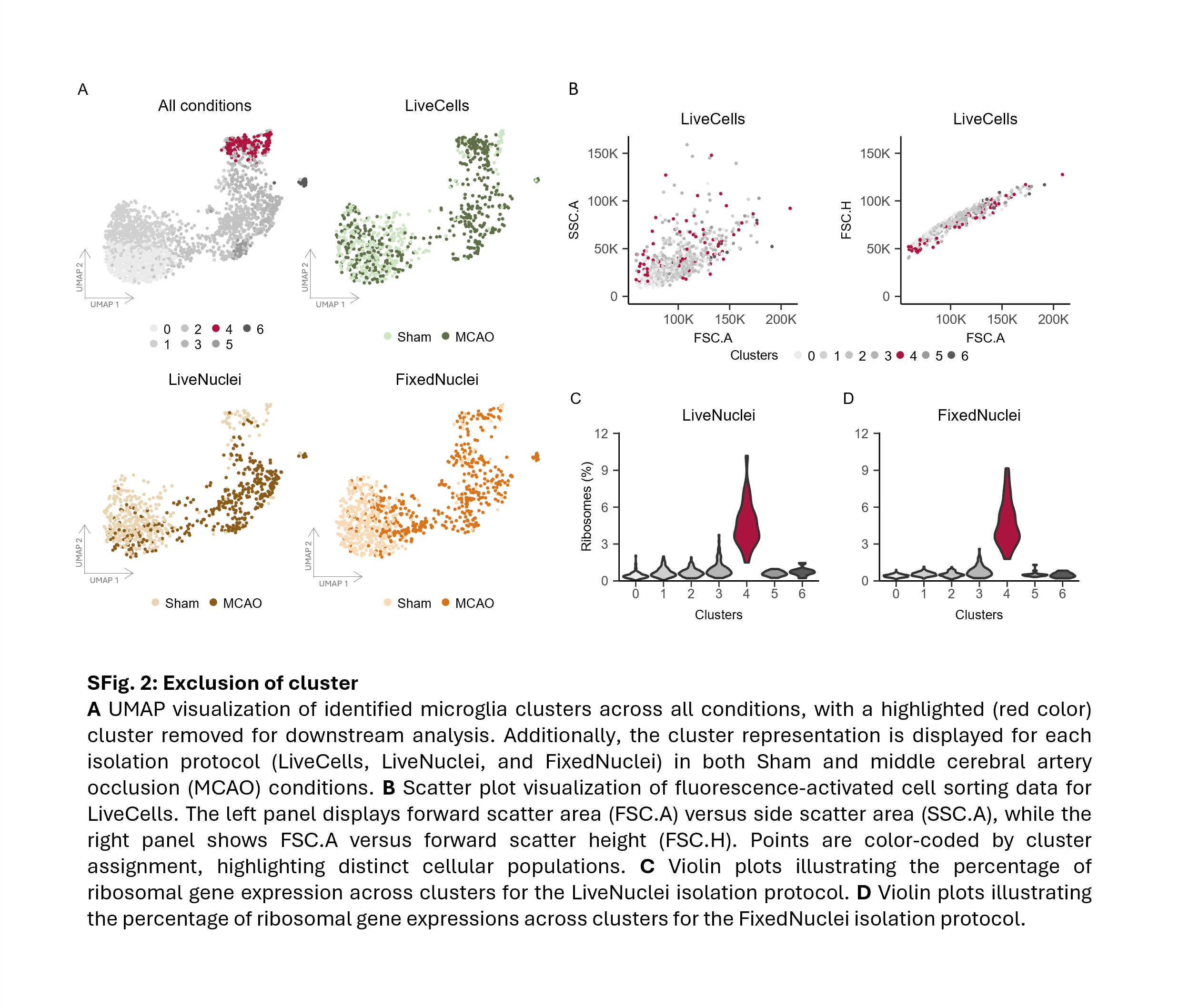

Supplement: Supplementary file 2 — Supplementary material 2 (ZIP 4520.9 kb) [file 10571_2026_1743_MOESM2_ESM.zip › SFig. 1_5/SFig. 2.tif]

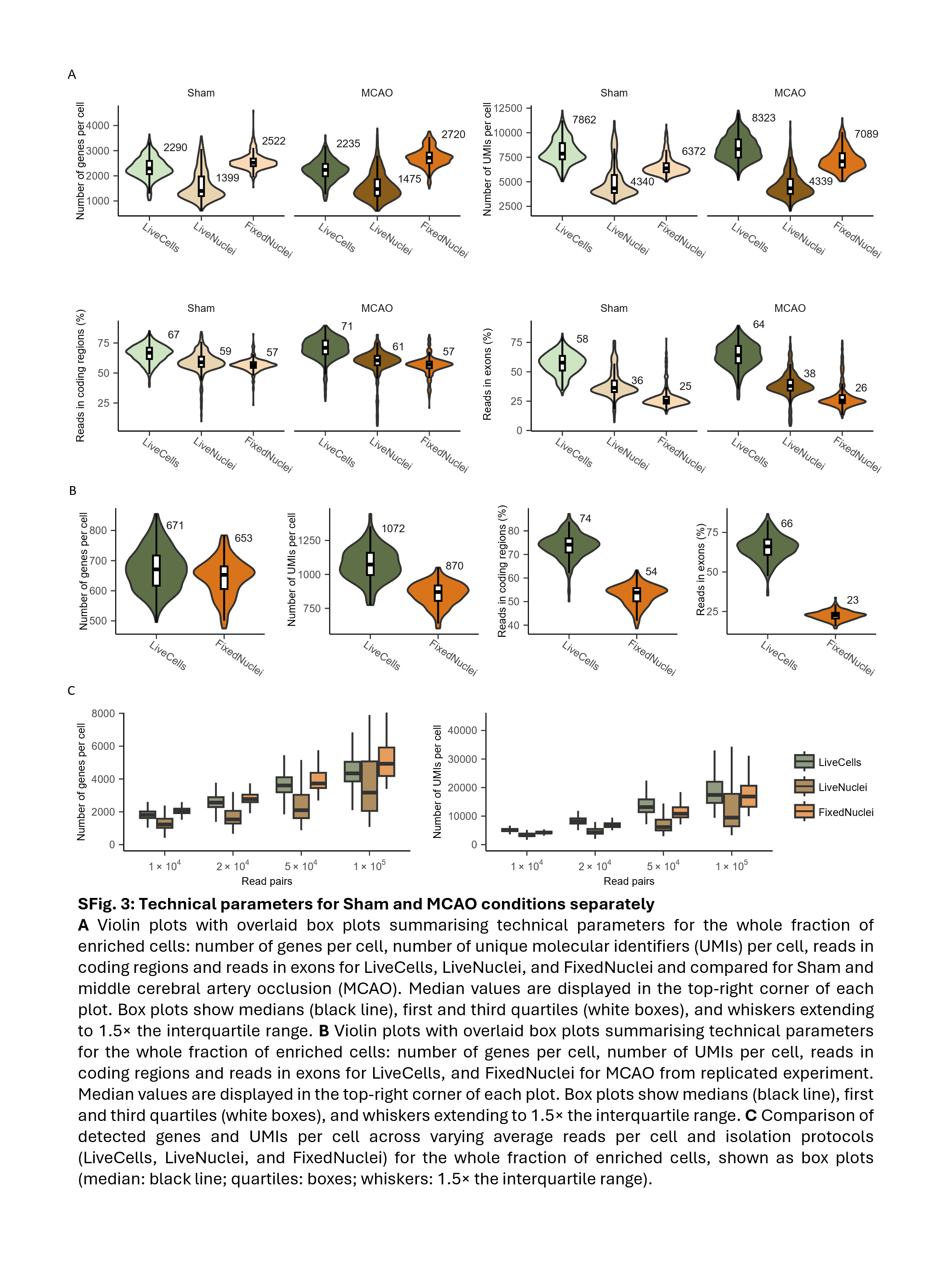

Supplement: Supplementary file 2 — Supplementary material 2 (ZIP 4520.9 kb) [file 10571_2026_1743_MOESM2_ESM.zip › SFig. 1_5/SFig. 3.tif]

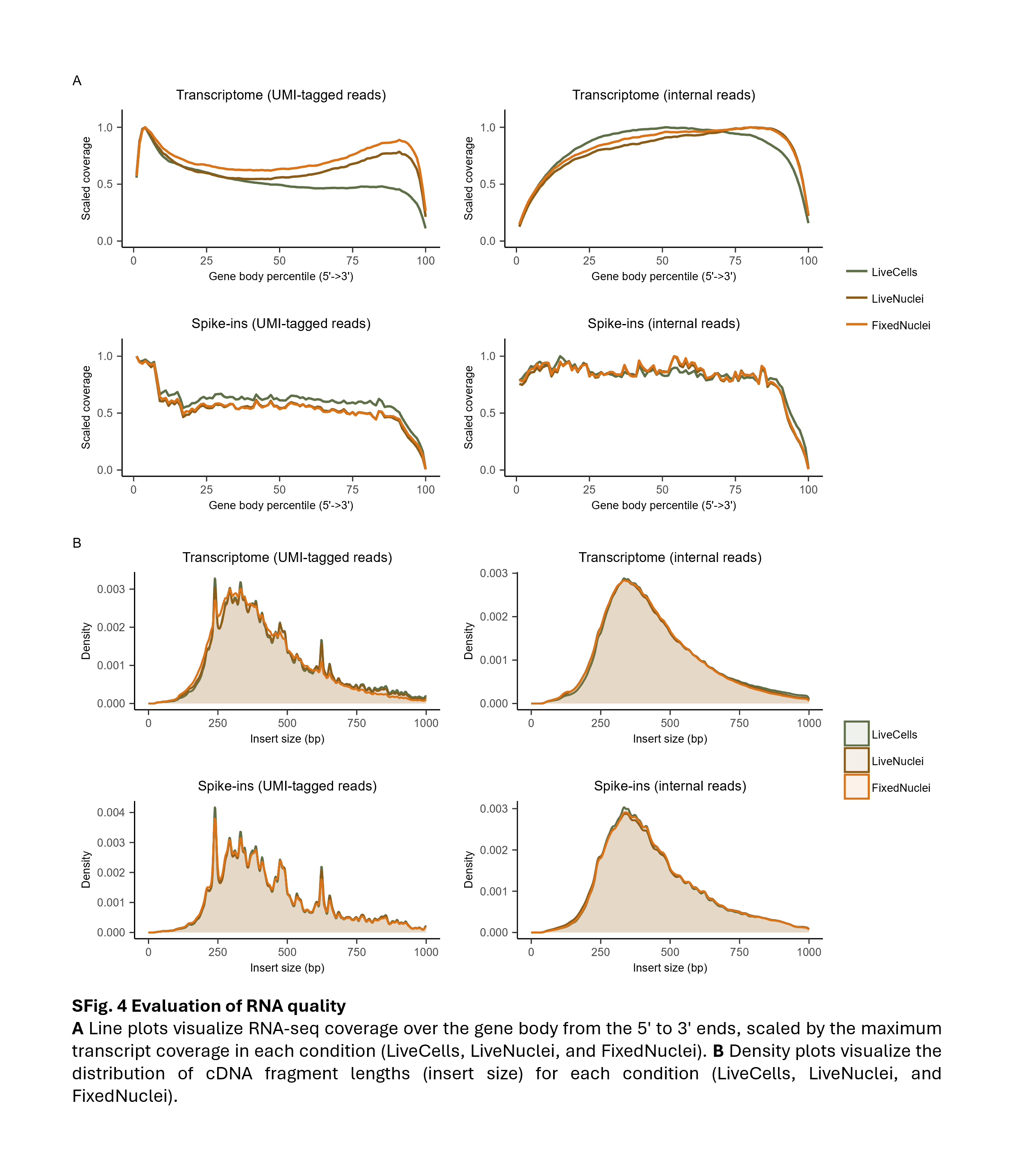

Supplement: Supplementary file 2 — Supplementary material 2 (ZIP 4520.9 kb) [file 10571_2026_1743_MOESM2_ESM.zip › SFig. 1_5/SFig. 4.tif]

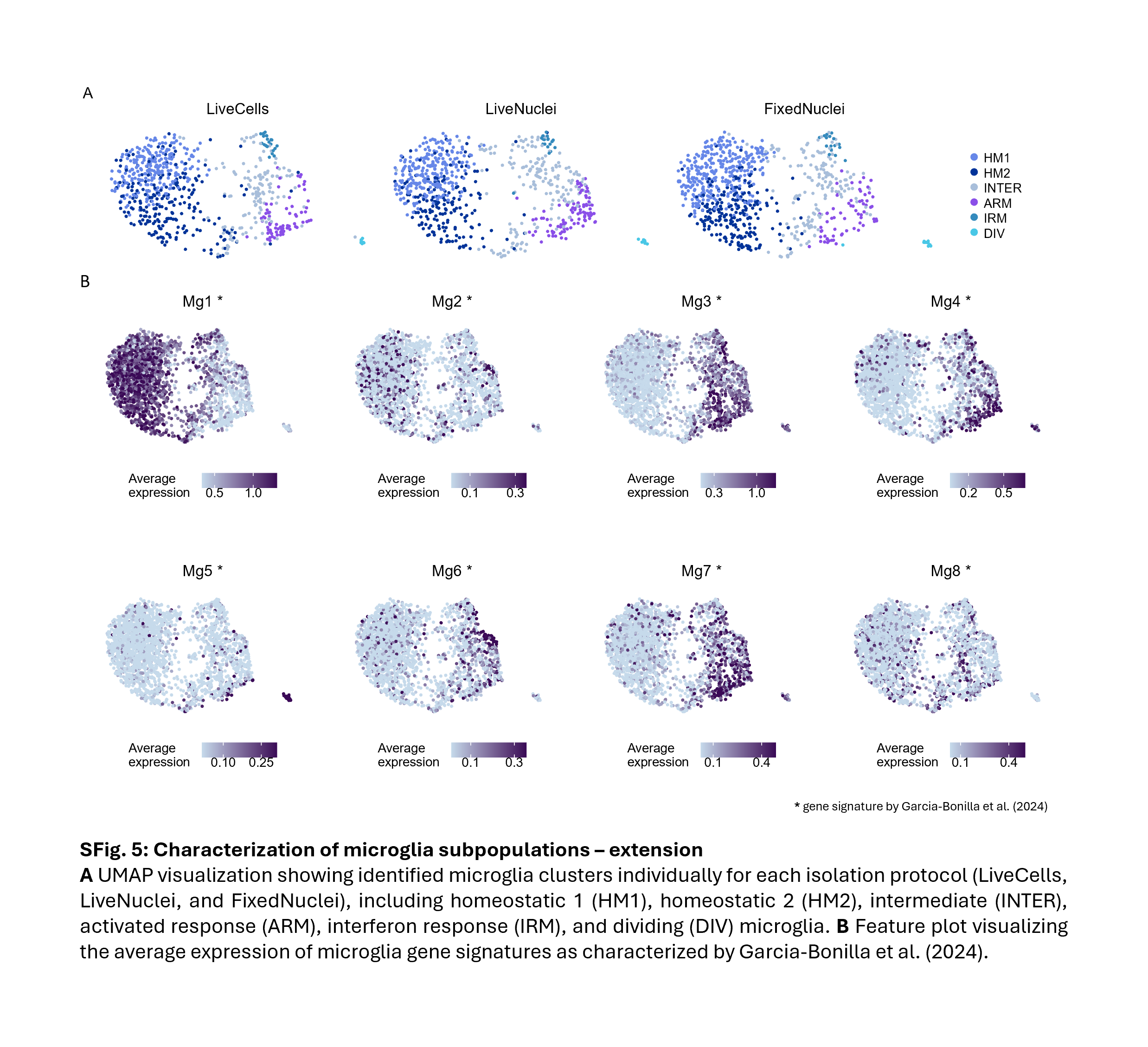

Supplement: Supplementary file 2 — Supplementary material 2 (ZIP 4520.9 kb) [file 10571_2026_1743_MOESM2_ESM.zip › SFig. 1_5/SFig. 5.tif]
